# V-ATPase controls tumor growth and autophagy in a Drosophila model of gliomagenesis

**DOI:** 10.1080/15548627.2021.1918915

**Published:** 2021-05-12

**Authors:** Miriam Formica, Alessandra Maria Storaci, Irene Bertolini, Francesca Carminati, Helene Knævelsrud, Valentina Vaira, Thomas Vaccari

**Affiliations:** a Department of Biosciences, Università Degli Studi Di Milano, Milan, Italy; b Division of Pathology, Fondazione IRCCS Ca’ Granda Ospedale Maggiore Policlinico, Milan, Italy; c Department of Pathophysiology and Transplantation, Università Degli Studi Di Milano, Milan, Italy; d Department of Molecular Cell Biology, Institute for Cancer Research, Oslo University Hospital, the Norwegian Radium Hospital, Oslo, Norway; e Centre for Cancer Cell Reprogramming, Institute of Clinical Medicine, Faculty of Medicine, University of Oslo, Oslo, Norway

**Keywords:** Autophagy, cancer model, fruit fly, glioblastoma, lysosomes, neurospheres, ref(2)P, V-ATPase

## Abstract

Glioblastoma (GBM), a very aggressive and incurable tumor, often results from constitutive activation of EGFR (epidermal growth factor receptor) and of phosphoinositide 3-kinase (PI3K). To understand the role of autophagy in the pathogenesis of glial tumors *in vivo*, we used an established *Drosophila melanogaster* model of glioma based on overexpression in larval glial cells of an active human *EGFR* and of the PI3K homolog *Pi3K92E/Dp110*. Interestingly, the resulting hyperplastic glia express high levels of key components of the lysosomal-autophagic compartment, including vacuolar-type H^+^-ATPase (V-ATPase) subunits and ref(2)P (refractory to Sigma P), the *Drosophila* homolog of SQSTM1/p62. However, cellular clearance of autophagic cargoes appears inhibited upstream of autophagosome formation. Remarkably, downregulation of subunits of V-ATPase, of *Pdk1*, or of the Tor (Target of rapamycin) complex 1 (TORC1) component *raptor* prevents overgrowth and normalize ref(2)P levels. In addition, downregulation of the V-ATPase subunit *VhaPPA1-1* reduces Akt and Tor-dependent signaling and restores clearance. Consistent with evidence in flies, neurospheres from patients with high V-ATPase subunit expression show inhibition of autophagy. Altogether, our data suggest that autophagy is repressed during glial tumorigenesis and that V-ATPase and MTORC1 components acting at lysosomes could represent therapeutic targets against GBM.

## Introduction

Gliomas, the most common brain malignancy, represent a challenge for therapy because of limited treatment options and of the onset of therapeutic resistance. Among gliomas, GBM is by far the most aggressive and incurable [1], with a 5-year survival rate of only 5% [2]. Even in patients with positive prognostic factors, maximum surgical resection and adjuvant chemoradiotherapy, the overall median survival rate is limited to 14.6 months [3]. The most frequent genetic feature of GBM is mutation of *EGFR*, leading to a constitutively activated form of the receptor in around 40–50% of primary GBMs [4]. The PI3K pathway, which is one of the EGFR effectors, can also be mutated in 20% of tumors, contributing to uncontrolled cell growth [5]. Thus, components of the EGFR and PI3K pathways, including the serine/threonine kinase Akt and the MTOR (mechanistic target of rapamycin kinase), are widely considered potential targets to develop new GBM treatments in combination with other therapeutics [6,7].

A recently discovered prognostic feature of GBM is the expression of subunits of the V-ATPase proton pump, which are frequently found upregulated in cancer [8,9]. In fact, in GBM tissue samples and GBM patient-derived neurospheres (NS), increased expression of a subset of V-ATPase subunits positively correlates with GBM aggressiveness and poor patient survival [10,11]. Interestingly, V-ATPase and the MTOR complex 1 (MTORC1) kinase act with the TFEB (transcription factor EB) family of lysosomal-associated proteins to form a homeostatic circuit that balances catabolic and anabolic processes [12,13]. When MTORC1 is inactive, TFEB translocates into the nucleus to modulate expression of genes harboring a coordinated lysosomal expression and regulation (CLEAR) site, thus controlling lysosomal biogenesis and macroautophagy (autophagy hereafter) [14]. However, whether and how V-ATPase regulates tumor growth in genetic models of glioma development is not known.

Cell type-specific regulation, genetic alterations, tumor staging or treatment most likely determine the exact role of autophagy in tumorigenesis [15,16]. For instance, during tumor initiation autophagy has been shown to play a tumor-suppressive role. However, once the tumor is established, autophagy can instead positively impact tumor survival by increasing metabolic activity in support of cell proliferation or survival to hypoxia [17]. Importantly, autophagy appears to play different roles in cancer stem cells, compared to differentiated cells, and provides resistance to chemotherapy [18–21]. In GBM, treatment with the drug temozolomide (TMZ) has been reported to trigger autophagy [22,23] and combination therapy with the V-ATPase inhibitor bafilomycin A_1_ (BafA1) increases glioma cell death [10,24]. Despite this, the role of autophagy in gliomagenesis remains largely underexplored.

In this study, we used *Drosophila melanogaster* as an *in vivo* model to define the role of V-ATPase and autophagy during glioma development. *Drosophila* encodes a single homolog of most genes altered in GBM, all displaying high degrees of functional conservation with mammals [25]. Our data indicate that autophagy is repressed both *in vivo* and in patient-derived NS and that V-ATPase, as well as components of the Akt-MTOR pathway, are likely limiting factors for growth and autophagy inhibition.

## Results and discussion

**Figure 1.**
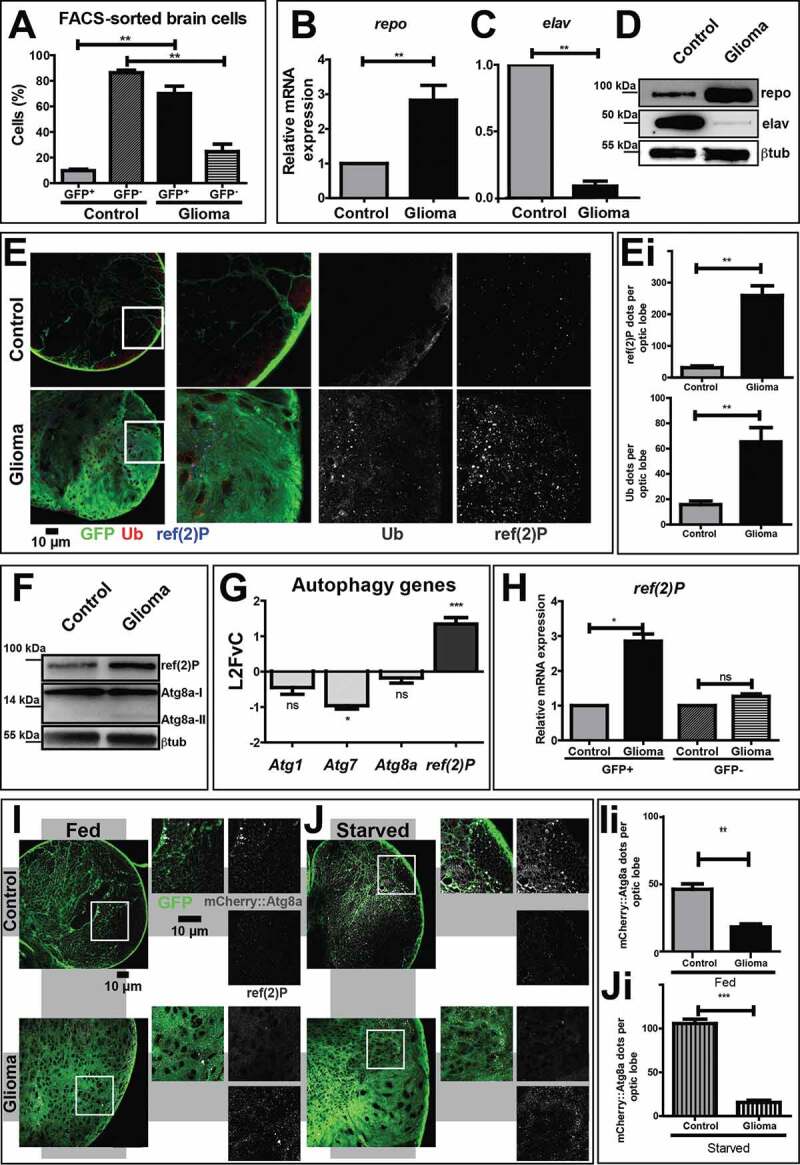
The autophagy-lysosomal pathway is inhibited during glial overgrowth induced by expression of *Pi3K92E* and *ΔhEGFR*. (A) Larval brain cells were separated by FACS. In controls, glial cells (GFP^+^) represent 10% of the total brain population, while, in gliomas, glial cells are up to 70%. Accordingly, glia overgrowth involves a strong reduction of neurons (GFP^−^) which are heavily decreased compared to control brains. Data represent the mean ± S.D. and *P‐values* are determined by Kruskal Wallis test with Dunn’s Multiple Comparison. (B and C) mRNA expression of *repo* (glia) and *elav* (neurons). *repo* levels are strongly increased in gliomas compared to controls. Conversely, *elav* levels are heavily suppressed in gliomas samples. *RpL32* is used as a housekeeping control. Data represent the mean ± S.D. and *P‐values* are determined by Mann-Whitney test. (D) Western blot showing repo and elav protein levels. repo levels confirm a strong expansion of glial tissue in gliomas, while elav protein levels are strongly decreased in tumor brains. βtub is used as a loading control. (E) Single medial confocal sections of third instar larval brains. High magnification insets are shown as merge and separate channels. Glial cell membranes (marked with anti-GFP), ubiquitin (marked by anti-ubiquitin FK2), ref(2)P are pseudo-colored as indicated. Notice the increased signal of both markers in gliomas compared to controls (quantified in Ei). Data represent the mean ± S.D. and *P‐values* are determined by t-test. Ubiquitin mostly colocalizes with ref(2)P. (F) Western blot showing expression of the autophagy markers ref(2)P and Atg8a I–II. In gliomas, ref(2)P protein levels are increased compared to controls, albeit Atg8a levels are only slightly changed. βtub is used as a loading control. (G) mRNA levels showing expression of autophagy genes. *ref(2)P* mRNA expression in glioma samples is strongly upregulated, while mRNA level of *Atg1, Atg7* and *Atg8a* are comparable to controls. Data are expressed as fold increase relative to control brains (L2FvC). Data represent the mean ± S.D. and *P‐values* are determined by Mann-Whitney test. (H) *ref(2)P* expression levels in GFP^+^ and GFP^−^ brains cells separated by FACS. *ref(2)P* levels are increased in GFP^+^ cells that belong to tumor tissue, but not in GFP^−^ neural cells. Normalization on GFP^+^ cells of control brains. *RpL32* is used as housekeeping. Data represent the mean ± S.D. and *P‐values* are determined by one-way ANOVA, Kruskal Wallis test with Dunn’s Multiple Comparison. (I and J) Single medial confocal sections of third instar larval brains reared under fed and starved conditions. Glial cell membranes are marked by GFP, Atg8a is detected by mCherry (mCherry::Atg8a). Note the strong increase of mCherry::Atg8a signal upon starvation in controls but not in gliomas. In contrast, upon starvation of gliomas ref(2)P is strongly accumulated (quantified in Ii and Ji). Data represent the mean ± S.D. and *P‐values* are determined by Mann-Whitney test

As previously demonstrated [25,26], co-expression in *Drosophila* larval glial cells of the constitutively active form of PI3K (*Pi3K92E-CAAX)* and of human *EGFR (ΔhEGFR)*, under the control of *repo-Gal4*, a *P(Gal4)* insertion in the glial-specific *reverse polarity* (*repo*) locus, promoted excess cell growth and hypertrophy of the optic lobes of the central nervous system (CNS) (Fig. S1A-Aiii). In control brains, glial cells constituted 10% of the CNS cells, while the rest was mostly formed by neurons and neural progenitors (Figure 1A [27];). Upon co-expression of *Pi3K92E-CAAX* and *ΔhEGFR*, cells of glial origin made up 70% of the recovered CNS cells (Figure 1A). Consistent with this, *repo* transcription was upregulated compared to controls, while expression of the neuronal marker *elav* (embryonic lethal abnormal vision) was strongly downregulated (Figure 1B and Figure 1C). Alongside, the corresponding repo and elav proteins were similarly deregulated (Figure 1D). Morphologically, larvae carrying gliomas showed an extremely altered CNS arrangement, with neurons located in small clusters surrounded by hyperplastic glia (Fig. S1B-Bi), and failed to wander or pupariate, eventually dying at third instar (Fig. S1C-Cii). These data confirm that the cell growth aspects of gliomagenesis can be recapitulated in *Drosophila* and extend the description of such *in vivo* genetic model.

To characterize the role of the autophagy-lysosomal pathway in gliomagenesis *in vivo*, we evaluated the presence of ubiquitin and the autophagy-specific cargo receptor ref(2)P in larval brains. Compared to control glia, in which very little signal of either marker was detected, both proteins strongly accumulate in puncta, often colocalizing within glial cells of larvae carrying gliomas (Figure 1E, quantified in Ei). This result suggests that during gliomagenesis, the autophagic process could be either impaired or heavily induced. To discriminate, we assessed the autophagic flux by monitoring the expression of ref(2)P and Atg8a (autophagy-related protein 8a; LC3 in mammals). We confirmed that ref(2)P accumulated in glioma CNS extracts, while Atg8a levels were only slightly increased when compared to control extracts (Figure 1F, quantified in Fig. S1D). In addition, we found that transcription of *Atg8a* and other core autophagy genes, such as *Atg1* and *Atg7* was mostly unchanged relative to controls, while that of *ref(2)P* was upregulated (Figure 1G). Then, we sorted brain cells to reveal that *ref(2)P* expression was increased exclusively in GFP^+^ glial cells belonging to tumor brains (Figure 1H). We next used starvation, a known inducer of autophagy, to evaluate whether such a pathway could be induced during gliomagenesis. Consistent with basal levels of constitutive autophagy, mCherry::Atg8a, a subcellular marker of autolysosome formation, could be detected in control glial tissue but not in glioma cells under fed condition (Figure 1I, quantified in 1Ii). Upon starvation, the mCherry::Atg8a signal was strongly increased in control samples, indicating induction of autolysosome formation by nutrient deprivation (Figure 1J, quantified in 1 Ji). In stark contrast, the CNS of larvae carrying gliomas showed no appreciable increment of mCherry::Atg8a level (Figure 1J, quantified in 1 Ji). Western blot to detect ref(2)P confirmed that its accumulation decreased during starvation in control, while it remained unchanged in glioma samples (Fig. S1E, quantified in S1F), indicating the inability to clear autophagic cargoes by autophagy.

**Figure 2.**
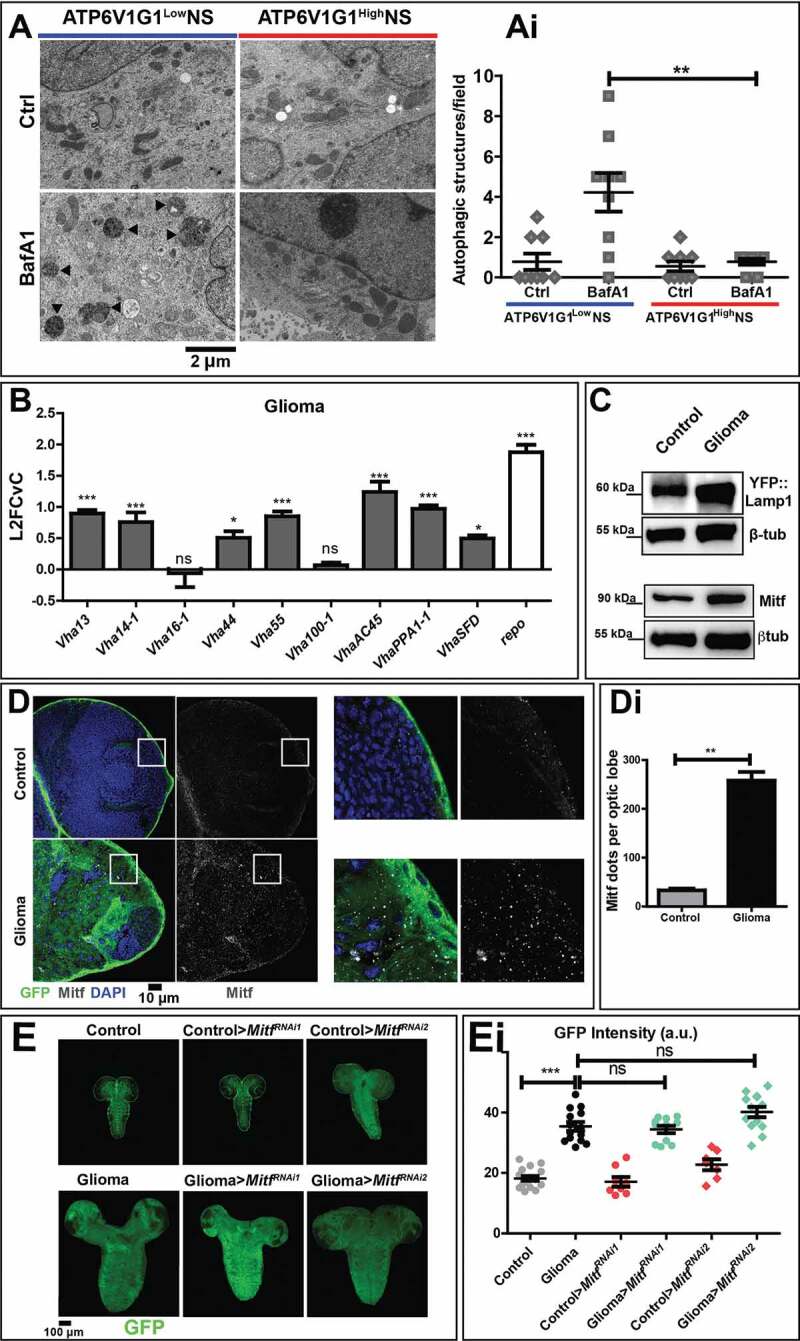
Autophagy in NS and characterization of the lysosomal compartment during gliomagenesis. (A) Representative EM images of NS treated with vehicle (Ctrl) and BafA1. ATP6V1G1^High^ and ATP6V1G1^Low^ NS show different accumulation of autophagic organelles (arrowheads) upon treatment. (Ai) Quantification of autophagic structures confirms that BafA1 causes accumulation of aberrant organelles only in ATP6V1G1^Low^ NS. Data represent the mean ± S.D. and *P‐value* is obtained by one-way ANOVA, Kruskal Wallis test with Dunn’s Multiple Comparison. (B) qPCR analysis of the indicated V-ATPase subunits and of *repo* in fly glioma cells relative to control. mRNA expression levels confirm the upregulation of the V-ATPase subunits in fly gliomas. Data are expressed as L2FCvC. Bars, mean ± S.D. and *P‐values* are determined by two-way ANOVA with Bonferroni correction. (C) Western blot showing expression of YFP::Lamp1 (top) and Mitf (bottom). In gliomas, levels of both proteins are increased compared to controls. βtub is used as a loading control. (D) Single medial confocal sections of third instar larval brains. Nuclei were stained with DAPI, glial cell membranes with anti-GFP. Mitf is heavily accumulated in glioma compared to control optic lobes (quantified in Di). Data represent the mean ± S.D. and *P‐values* are determined by Mann-Whitney test. Mitf accumulation can be better appreciated in higher magnifications of insets. Notice that in gliomas, Mitf is almost exclusively in the cytoplasm, not in nuclei (insets). (E) Single medial confocal section of whole CNS of third instar larvae. Dorsal view, anterior up. Larval brains carrying gliomas in which *Mitf* has been downregulated show similar growth to gliomas(Quantified in Ei). Mean ± SEM and *P*‐*values* are determined by one-way ANOVA, Kruskal Wallis test with Dunn’s Multiple Comparison

To test whether autophagy is also inhibited in patient-derived GBM NS, we examined the morphology of degradative organelles by electron microscopy. We briefly treated NS with the V-ATPase inhibitor BafA1 that blocks fusion of autophagosomes to lysosomes [28], and quantified the number of autophagosomes, which is expected to accumulate upon treatment only in NS with active autophagy. We found that in NS with a low level of ATP6V1G1 (ATPase H+ transporting V1 subunit G1) subunit expression (ATP6V1G1^Low^NS), BafA1 administration led to major accumulation of autophagic structures (Figure 2A). In contrast, in NS from patients with elevated expression of ATP6V1G1 (ATP6V1G1^High^NS), drug treatment did not significantly change the number of autophagic structures, which was comparable to untreated controls (Figure 2A, quantified in Ai). Overall, these findings suggest that in both fly gliomas and patient-derived NS with high ATP6V1G1 expression, autophagy is inhibited upstream of autophagosome formation.

Since autophagy and V-ATPase are part of lysosomal nutrient-regulation circuits, we next assessed V-ATPase subunit expression, lysosome abundance and function in cells undergoing gliomagenesis in *Drosophila*. Interestingly, we found that 7 out of the 9 V-ATPase subunits tested, namely *Vha13* (encoding the *Drosophila* subunit V1G), *Vha14-1* (V1F), *Vha44* (V1C), *Vha55* (V1B), *VhaAC45* (V0AC45), *VhaPPA1-1* (V0B) and *VhaSFD* (V1H) were highly expressed in larvae carrying gliomas, when compared to controls (Figure 2B), echoing the elevated expression observed in aggressive GBM NS. FACS analysis confirmed that expression of *VhaPPA1-1* and *Vha13 wa*s higher in glial cells than in other CNS cell types (Fig. S2A and S2B), as suggested by previous evidence on glial functions [27,29]. Protein and mRNA expression of Lamp1 (lysosomal-associated membrane protein 1) was also elevated in glial tumor cells (Figure 2C and S2C). Similarly, expression of Mitf (microphthalmia-associated transcription factor), the unique fly TFEB homolog [30–32], was increased in gliomas compared to controls (Figure 2C and S2D). However, immunofluorescence analysis revealed that Mitf is not appreciably present in the nuclei of glioma cells, compared to those of control glia, suggesting that its increase does not correlate with increased activity (Figure 2D, quantified in Di). In agreement with this observation, the degradative ability of lysosomes, measured by DQ-bovine serum albumin (BSA) uptake (see Material and Methods) was preserved, if not increased, in the CNS of larvae carrying gliomas (Fig. S2E, quantified in Ei). These data demonstrate that during fly gliomagenesis the lysosomal compartment of glial cell is moderately expanded and active, while TFEB is mostly inactive and not contributing to tumor growth.

**Figure 3.**
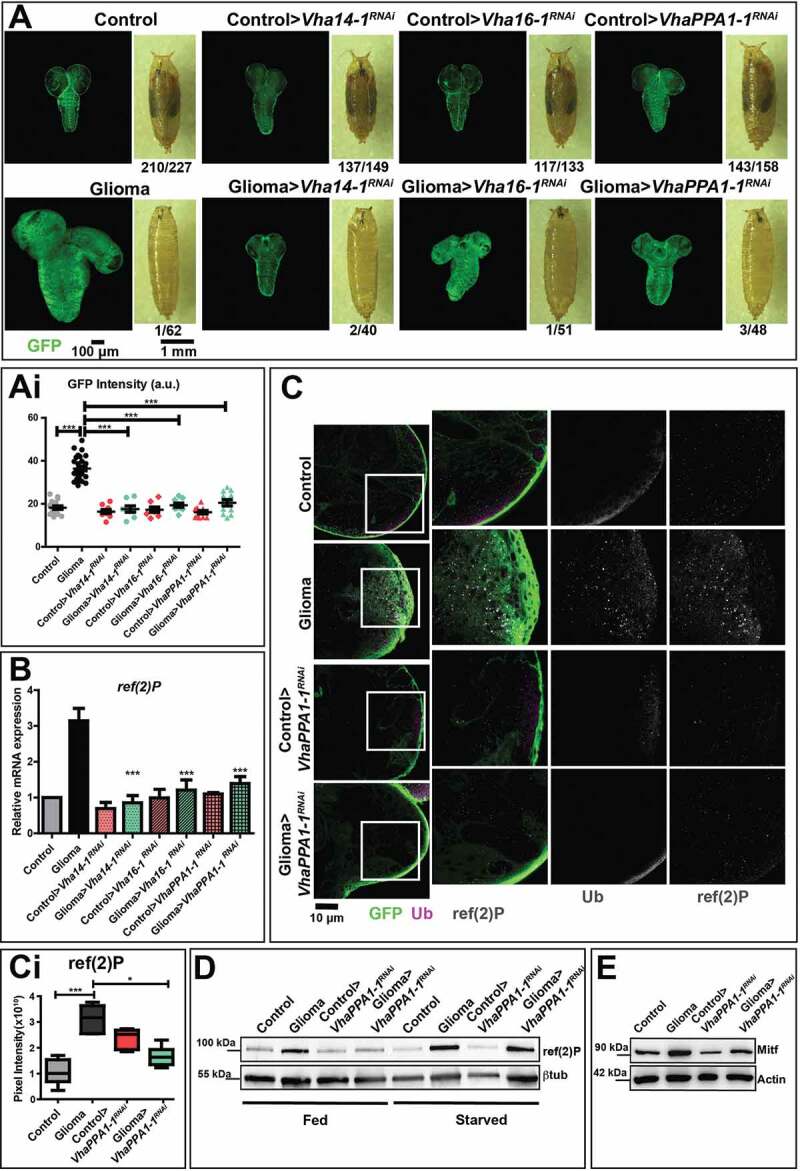
Downregulation of V-ATPase subunits restores normal growth and autophagy in cells subjected to gliomagenesis. (A) Single medial confocal sections of a whole CNS from third instar larvae. Dorsal view, anterior up. The excess growth of the glia, observed in gliomas, is reduced to control levels by the indicated depletions (quantified in Ai). Mean ± S.D. are shown, and *P*‐values are determined by one-way ANOVA, Kruskal Wallis test with Dunn’s Multiple Comparison. Example of pupae of the indicated genotypes is shown to the right of the CNS images (quantification below each panel shows the number of pupae reaching metamorphosis over the total). (B) *ref(2)P* mRNA levels by qPCR. Data represent the mean ± S.D. of n ≥ 3 independent experiments and *P*‐*values* are obtained by Kruskal Wallis test with Dunn’s Multiple Comparison. (C) Single medial confocal sections of third instar larval CNSs immunostained as indicated. The increased ubiquitin (Ub) and ref(2)P signal observed in gliomas, is absent in gliomas>*VhaPPA1-1^RNA^*^i^. See also insets on the right of each panel. (Quantification in Ci). Mean ± S.D are shown, *P*‐*values* are determined by one-way ANOVA, Kruskal Wallis test with Dunn’s Multiple Comparison. (D and E) Western blot showing levels of ref(2)P (D) or Mitf (E) in the indicated conditions. βtub (D) or Actin (E) are used as a loading control

Spurred by the elevated expression of certain V-ATPase subunits in fly gliomas and in patient-derived NS, we next examined whether the hyperplastic glial growth depends on V-ATPase subunits expression. Interestingly, *Mitf* downregulation during gliomagenesis led to a 2-4-folds reduction of expression of *Lamp1* as well as all the V-ATPase subunits tested with the exception of *Vha100-1, Vha13* and *Vha44*, suggesting that the upregulation observed during gliomagenesis depends, at least in part, on Mitf activity (Fig. S2D and S2F). However, *Mitf* downregulation did not appear *per se* sufficient to affect glioma growth (Figure 2E, quantification in Ei). In contrast, approximately 6-8-fold individual downregulation in the context of *Drosophila* gliomagenesis of specifically the V-ATPase subunits *Vha14-1, Vha16-1* or *VhaPPA1-1* prevented glial cell overgrowth. However, it did not rescue progression to metamorphosis (Figure 3A, quantified in Ai; Fig. S3A [11],). These data reveal that gliomagenesis is in part prevented by limiting the expression of certain V-ATPase subunits.

To study glioma tissue development with limiting V-ATPase expression, we analyzed more in detail glial-specific *VhaPPA1-1* downregulation. In addition to reduced glial overgrowth, here we found that elav protein levels are increased in gliomas>*VhaPPA1-1 ^RNAi^*, when compared to glioma CNSs, indicating a partial reversion of the neuronal loss induced by gliomagenesis (Fig. S3B). Despite this, alteration of CNS architecture was not rescued by V-ATPase subunit downregulation (Fig. S3C), suggesting that not all aspects of gliomagenesis are reverted by *VhaPPA1-1* subunit downregulation. To investigate whether glial-specific downregulation of *VhaPPA1-1* restricts growth by causing cell death, we evaluated tissue expression of the apoptotic marker cleaved Decay/caspase 3. Unexpectedly, we found that in controls>*VhaPPA1-1^RNAi^* apoptosis was strongly induced in both glia and neurons, consistent with the possibility that V-ATPase is essential autonomously and non-autonomously for CNS health (Fig. S3C, quantified in Ci). However, this was not the case in larvae carrying gliomas (Fig. S3C, quantified in Ci), which we previously found to contain higher glial V-ATPase subunit expression than in healthy larvae (Figure 2B, S2A and S2B). This result reveals that VhaPPA1-1 is essential for survival of otherwise wild-type glial cells, but not for the survival of overgrowing glia.

To uncover the mechanism that underlies the dependency of glioma growth on V-ATPase, we first assessed *ref(2)P* expression. Notably, the upregulation of *ref(2)P* at the mRNA level observed in gliomas was reverted upon downregulation of *VhaPPA1-1, Vha14-1* and *Vha16-1* (Figure 3B). Consistent with this, but opposite to the strong ubiquitin and ref(2)P accumulation observed in glioma tissue, neither markers were found as puncta in tumor tissue depleted of *VhaPPA1-1* (Figure 3C; quantified in 3 Ci). In addition, the upregulation of ref(2)P observed in gliomas upon starvation was reverted upon *VhaPPA1-1* subunit downregulation (Figure 3D). Also, Mitf accumulation in glioma CNSs was prevented by downregulation of *VhaPPA1-1* (Figure 3E and S3D, quantified in Di). Finally, lysosomal activity was not altered following *VhaPPA1-1* downregulation, while the partial expansion of the lysosomal compartment observed in glioma CNSs was reverted (Fig. S3E, quantified in Ei), suggesting that V-ATPase downmodulation might normalize the alterations of catabolism associated to fly gliomagenesis.

**Figure 4.**
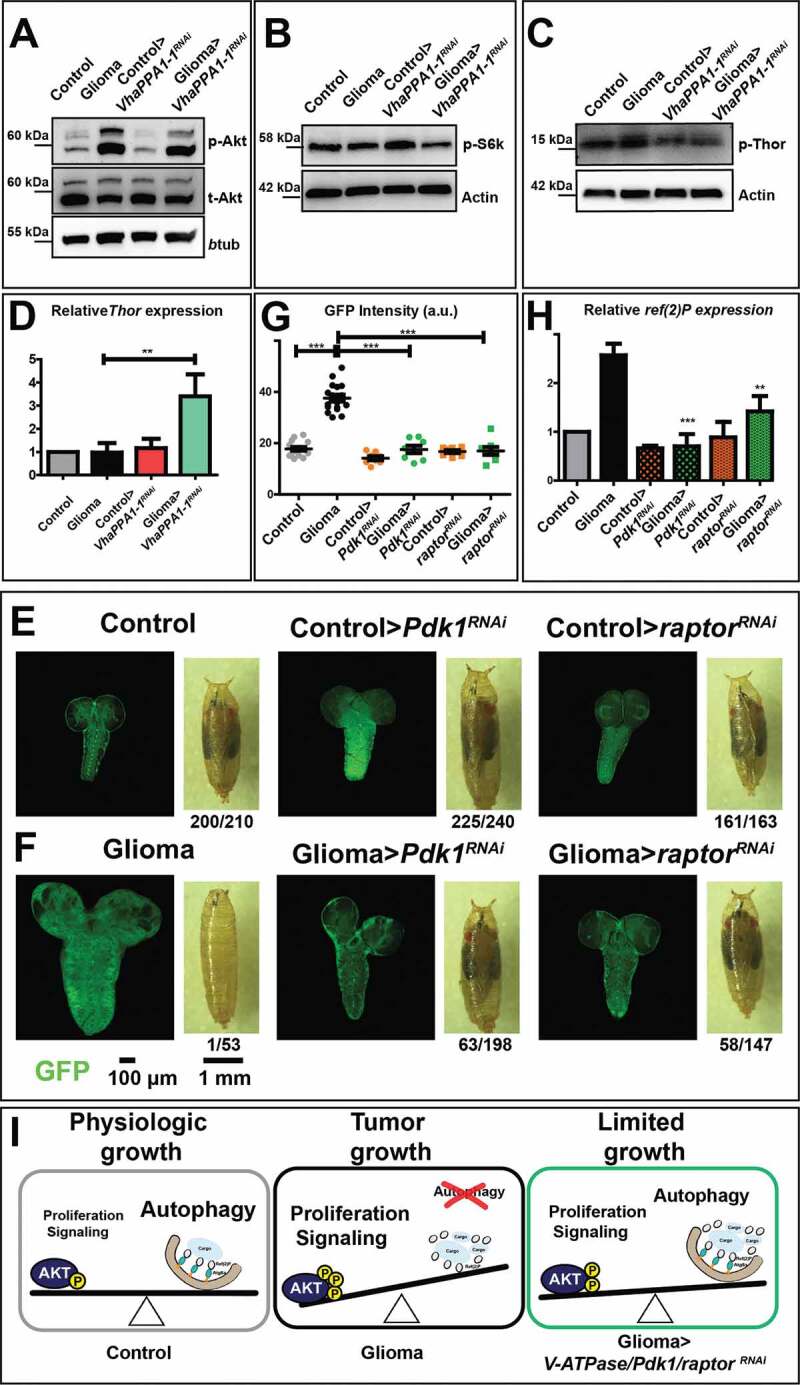
Effects on Akt signaling and gliomagenesis upon downregulation of *VhaPPA1-1, Pdk1* or *raptor*. (A) Western blot showing Akt and phosphorylated Akt (p-Akt) levels. The increased p-Akt levels observed in gliomas are decreased upon *VhaPPA1-1* downregulation. Total Akt (t-Akt) and βtub levels are used as loading control. (B and C) Western blot showing phosphorylated S6k (p-S6k; B) or phosphorylated Thor (p-Thor; C) levels. p-S6k or p-Thor levels in gliomas are decreased upon *VhaPPA1-1* downregulation. Actin levels are used as loading control. (D) *Thor* mRNA levels by qPCR are upregulated in gliomas>*VhaPPA1-1^RNAi^*. Expression levels are relative to control brains. Data represent the mean ± S.D. and *P*‐*value* was obtained by one-way analysis of variance, Bonferroni’s Multiple Comparison Test. (E and F) Single medial confocal sections of a whole CNS from third instar larvae. Dorsal view, anterior up. The excess growth of the glia, observed in gliomas, is reduced to control levels by the indicated depletions (quantified in G). Mean ± S.D. and *P*‐values are determined by one-way ANOVA, Kruskal Wallis test with Dunn’s Multiple Comparison. Example of pupae of the indicated genotypes is shown to the right of the CNS images (quantification below each panel shows the number of pupae reaching metamorphosis over the total). (H) *ref(2)P* mRNA levels by qPCR. Data represent the mean ± S.D. and *P*‐*values* are obtained by one-way ANOVA, Kruskal Wallis test with Dunn’s Multiple Comparison. (I) A model for V-ATPase function in *Drosophila* larval gliomas. The physiologic balance between anabolic and catabolic processes governing normal cell growth (Physiologic growth) is heavily compromised in gliomas. Indeed, in tumor brains, growth is enhanced while catabolism is impaired (Tumor growth). Downregulation of *VhaPPA1-1, Pdk1* or *raptor* restore the equilibrium controlling nutrient metabolism, ultimately derepressing autophagy and decreasing tumor growth (Limited growth)

We next evaluated the activation of growth signaling by detecting phosphorylation of *Drosophila* Akt (p-Akt). As expected, we found a sharp increase in p-Akt levels in glioma samples. Importantly, such an increase was blunted by downregulation of *VhaPPA1-1* (Figure 4A, quantified in Fig. S4A). We then monitored the process of translation regulation which is downstream of Akt signaling. Phosphorylation of the *Drosophila* translational activator ribosomal protein S6k also appeared reduced upon *VhaPPA1-1* downregulation during gliomagenesis (Figure 4B, quantified in Fig. S4B). Similar to S6k phosphorylation, phosphorylation of *Drosophila* Thor, the homolog of EIF4EBP1, a translation repressor that is downregulated and hyper-phosphorylated by Akt/MTOR activity [33,34], was decreased upon *VhaPPA1-1^RNAi^* downregulation (Figure 4C, quantified in Fig. S4C). Consistent with this, we observed that expression of *Thor* was elevated in gliomas>*VhaPPA1-1^RNAi^* when compared to controls (Figure 4D). These data suggest that the level of *VhaPPA1-1* limits activation of Akt and downstream signaling pathways. In agreement with this possibility, glial-specific downregulation of *Drosophila Pdk1* (3-phosphoinositide-dependent protein kinase), a well-known Akt activator downstream of PI3K [35], fully prevented overgrowth (Figure 4E, quantified in G; Fig. S4D).

To test directly whether Akt contribution to growth is mediated by the MTOR pathway acting at lysosomes, we considered the MTORC1 component *raptor* [36]. Notably, expression of both *raptor* and *Pdk1* was not increased in gliomas (Fig. S4D), in sharp contrast with expression of V-ATPase components (Figure 2B), suggesting that their activity could be even more limiting than that of V-ATPase during gliomagenesis. Consistent with this, we observed that glial overgrowth was prevented by *raptor* downregulation to the same extent as by *Pdk1* downregulation (Figure 4E and Figure 4F, quantified in 4 G; Fig. S4D). Different than V-ATPase subunit downregulation (Figure 3A), both *Pdk1* and *raptor* downregulation during gliomagenesis allowed progression of larvae to metamorphosis (Figure 4E and Figure 4F). Finally, we observed, that as in the case of *VhaPPA1-1* downregulation, downregulation of either *Pdk1* or *raptor* restored *ref(2)P* levels to those observed in control animals (Figure 4H). Overall, these data suggest that reduction of activity of the V-ATPase/MTOR axis during gliomagenesis restores catabolism operated by the autophagy-lysosomal pathway and restrains activation of growth pathways promoted by excess PI3K signaling.

*Drosophila* models of tumorigenesis have so far shown that autophagy promotes tumor growth in cancer stem cells in the ovary [37], as well as in *Ras*-, but not in JNK- or Notch-induced tumors in imaginal discs [16,38]. While we have previously reported that downregulation of *VhaPPA1-1* prevents excess growth in a fly model of gliomagenesis [11], this study explores, for the first time, autophagy and the V-ATPase/TFEB axis in the model.

Compared to nutrient sensing in non-tumor cells (Figure 4I; Physiologic growth), our data revealed that ectopic activation of PI3K signaling might fuel cell growth by increasing anabolism at the expense of catabolism associated with autophagy activation. Whether this is the case, or whether inhibition of autophagy upstream of autophagosome formation – indicated by a prominent accumulation of ref(2)P and ubiquitinated cargoes, but not of Atg8a – represents merely a side effect of oncogenic proliferative signaling remains to be determined. Persistent growth signaling might also conflict with lysosomal sensing of available nutrients and/or with changes in nutrient demand experienced by overgrowing tumor cells, leading to the TFEB and lysosomal compartment anomalies that we have observed in gliomas. *ΔhEGFR* and *Pi3K92E-CAAX*-mediated activation of the PI3K/Akt/MTOR pathway could prevent TFEB-mediated transcriptional regulation of genes related to the lysosomal-autophagic pathway, and/or it could directly inhibit Atg1 activity. As recently reported, Akt could also repress TFEB activity in an MTORC1-independent manner [39]. Despite this, we found that V-ATPase expression is reduced by downregulation of *Mitf*, suggesting that TFEB circuits are still partly active during gliomagenesis and contribute to elevating V-ATPase expression transcriptionally (Figure 4I; Tumor growth). In such context, how could reduction of V-ATPase, *Pdk1* and *raptor* expression prevent glial overgrowth and normalize ref(2)P/SQSTM1 levels? Rather than preventing lysosomal degradation of autophagic cargoes, as observed upon loss of function mutants for V-ATPase subunit genes [40], we propose that downregulation of certain V-ATPase subunits or, even more efficiently, of *Pdk1* and *raptor*, reduce anabolism mediated by the MTOR pathway. This might rebalance perturbed lysosomal-associated nutrient sensing and catabolic processes, such as autophagy, ultimately limiting tissue growth (Figure 4I; Limited growth). In light of this, we propose that the V-ATPase-MTOR axis, acting at lysosomes, could be a sensitive node to control the equilibrium between anabolic and catabolic cellular processes in glial tumors.

Our fly model recapitulates aspects of human glioblastoma, including the following evidence obtained with mammalian models and patient samples: Elevated expression of V-ATPase subunits [10,11]; Induction of autophagy by Akt inhibitors in glioma cells [41]; Proliferation arrest induced by PI3K-MTOR dual inhibitors [42]; Reduction of tumor growth and induction of autophagy by downregulation of the PI3K-Akt-MTOR pathway [43]. Thus, we foresee that future study of fly gliomas could provide a framework to uncover new genetic vulnerabilities in GBM. In addition, our model might provide a valuable entry point to test *in vivo* the efficacy of inducers of autophagy and of other modulators of the V-ATPase-TFEB axis as growth inhibitors. Finally, because standard treatment of gliomas with TMZ induces autophagy and the combination of TMZ with BafA1 enhances cell death in glioma cells [22,44], future evidence obtained with the fly model could direct us to an informed development of TMZ-based combination therapies.

Despite the evolutionary distance, the ability to model many of the main alterations observed in GBM, as well as the possibility to genetically and pharmacologically interrogate the model, might prove an advantage over monogenic mammalian models. A case in point is that of a murine RAS-only model, which reported an increase in autophagy during gliomagenesis [21]. However, fly gliomas might not recapture complex aspects of GBM, such as the differences observed between glioma stem cells and other glioma cells in terms of regulation of autophagy [20]. Interestingly, an alternative genetic model of gliomagenesis in flies exists [45] and it could be used to verify outcomes of future experiments. Of note, accumulation of SQSTM1 in absence of autophagosome formation has also been observed in mice lacking ATG7, which have been reported to develop spontaneous liver tumors. Interestingly, in such a background SQSTM1 contributes to tumor progression [46]. Thus, it would be interesting to assess the role of SQSTM1 and uncleared cargoes in promoting gliomagenesis, as well as the effect of autophagy modulators during tumorigenesis in highly nutrient-sensitive tissues.

## Materials and methods

### Drosophila husbandry

Fly strains were kept and raised into vials containing standard yeast-cornmeal fly food medium. All crosses were performed at 25°C. *Drosophila* lines used in this study were provided by the Bloomington *Drosophila* Stock Center (BDSC, Bloomington, Indiana), the Vienna *Drosophila* Resource Center (VDRC, Vienna, Austria) and by the *Drosophila* Genomics and Genetic Resources (DGGR, Kyoto, Japan). *repo-Gal4, UAS-CD8GFP, UAS-Pi3K92E-CAAX, UAS-ΔhEGFR* lines were kindly provided by Renee Read (Emory University School of Medicine, Atlanta, Georgia) [26]; *3xmCherry::Atg8a* was provided by Gabor Juhasz (Eotvos Lorand University, Budapest, Hungary) [47]; *UAS-VhaPPA1-1[GD16478]^RNAi^* (VDRC, 47,188), *UAS-Vha14-[KK10297]1^RNAi^* (VDRC, 110,160), *UAS-Vha16-1[GD17431]^RNAi^* (VDRC, 49,291), *UAS-Mitf [KK113614]^RNAi1^* (VDRC, 108,519), *UAS-Mitf[TRIP-HMS02712]^RNAi2^* (BDSC, 43,998), *UAS-Pdk1[KK108363]^RNAi^* (VDRC, 109,812), *UAS-raptor[KK108260]^RNAi^* (VDRC, 106,491), and *YFP::Lamp1* (DGGR, CPTI-001775 [48]). To induce starvation, larvae were washed in PBS 1X to remove food residues and left for 4 h on a Petri dish containing sucrose 20% diluted in PBS 1X. After starvation, larvae were dissected to isolate brains for the subsequent analysis. To monitor lysosomal degradation *in vivo*, larvae were incubated with DQ-BSA (Sigma, D12051) for 6 h. All genotypes of the experiments are listed in Table S1.

### Immunostaining

Larval brains were fixed using 4% PFA (Thermo Fisher Scientific, 28,908). Tissues were permeabilized with PBST (1X PBS [Gibco, 18,912,014], 1% Triton X-100 [Calbiochem, 9002–93-1]). Samples were blocked for 30 min in 4% BSA (Euroclone, EMR086500) diluted in PBST at room temperature. Primary antibody was incubated overnight at 4°C. The secondary antibody was incubated at room temperature for 2 h. Primary antibodies and dilutions used were: Rabbit cleaved CASP3/Decay 1:200 (Cell Signaling Technologies, 9661), chicken anti-GFP 1:1000 (Abcam, ab13970), mouse anti-FK2 1:250 (Enzo, BML-PW8810), rabbit anti-ref(2)P 1:1000 (a gift from Tor Erik Rusten, Oslo University, Oslo, Norway) [49], rat anti-RFP 1:1000 (Chromotek, 5F8), rabbit anti-Mitf 1:200 (developed by our group [32]). Alexa Fluor-conjugated secondary antibodies and dilutions used were: anti-mouse 546 1:400 (Invitrogen, A11030), anti-rabbit 647 (Invitrogen, A32733), anti-rat 647 (Invitrogen, A48265), and anti-chicken 488 (Invitrogen, A11039). Samples were mounted on slides using glycerol 70% (Merck Life science, G5516). Confocal acquisitions were performed using Leica SP2 microscope (Heidelberg, Germany) with ×40/NA 1.25 or ×63/NA 1.4 oil lenses or A1R confocal microscope (Nikon) or a Zeiss LSM880 (Carl Zeiss) equipped with an Ar-laser multiline (458/488/514 nm) with x10/NA 0.45 lenses. Measurements and fluorescence evaluation were carried out through the ImageJ Software (National Institutes of Health, Bethesda, USA), images were assembled with Adobe Illustrator.

### qPCR analysis

Larval brains were collected and homogenized using pestles. RNA extraction was performed using RNeasy Mini Kit (Qiagen, 74,104). The concentration of extracted RNA was measured using the NanoDrop 1000 Spectrophotometer. Complementary DNA (cDNA) was synthesized from RNA through reverse transcription, according to the SuperScript® VILO™ cDNA Synthesis Kit (Invitrogen, 11,754,050). Real-time PCR was carried out on the ABI/Prism 7900 HT Sequence Detector System (Applied Biosystems, Carlsbad, CA, USA) using primers that were designed from Universal Probe Library (UPL) Roche. These reactions were performed by the Cogentech qPCR service facility (Milan, Italy). Alternatively, samples were analyzed using StepOnePlus™ Real-Time PCR Systems (Applied Biosystems, Carlsbad, CA, USA), Fast SYBR Green Master Mix (Thermo Fisher Scientific, 4,385,617) and primers selected from http://www.flyrnai.org/flyprimerbank [50]. Amplicon expression in each sample was normalized to *RpL32* mRNA content. Primers sequences are listed in Table S2.

### Western blot

*Drosophila* larval brains were homogenized with pestles in RIPA buffer plus the addition of proteinase inhibitors 1:200 (Calbiochem, 539,134) and phosphatase inhibitors (Roche, 04906837001). The homogenate was centrifuged at 20,000 g for 20 min at 4°C. The supernatant was collected and quantified to determine the concentration of proteins in the sample, through the use of Pierce Bicinchoninic Acid Assay (BCA) Protein Assay Kit (Thermo Fisher Scientific, 23,227) method. Proteins were denatured in the Laemmli buffer and boiled for 5 min at 98°C. Proteins were separated by SDS gel-electrophoresis, the membrane was incubated with 5% milk (Merck Life science, 70,166) or 5% BSA for 1 h at room temperature. Then, the primary antibody of interest was added for 2 h at room temperature or overnight at 4°C. After the incubation, the membrane was incubated with specific secondary antibodies for 1 h at room temperature. Immunoblots were visualized using SuperSignal West pico/femto Chemiluminescent Substrate (Thermo Fisher Scientific, 34,080–34,095) and Chemidoc (Bio-Rad, Hercules, CA, USA). Primary antibodies used were: Rabbit anti-ref(2)P 1:1000 (a gift from Tor Erik Rusten, Oslo University, Oslo, Norway) [49], rabbit anti-Atg8a 1:5000 (from Gabor Juhasz, Eotvos Lorand University, Budapest, Hungary) [51], mouse anti-TUBB 1:8000 (GE Healthcare, 13–8000), rabbit anti-ACTB 1:1000 (Abcam, ab8227), mouse anti-repo 1:20 (Developmental Studies Hybridoma Bank, 8D12), rat anti-elav 1:40 (Developmental Studies Hybridoma Bank, 7E8A10), rabbit anti-Mitf 1:200, rabbit anti-phospho-Akt (Ser473) 1:1000 (Cell Signaling Technology, 9271), rabbit anti-Akt 1:1000 (Cell Signaling Technology, 9272), rabbit anti-p-Thor 1:500 (Cell Signaling Technology, 2855), mouse anti-p-S6k 1:300 (Cell signaling Technology, 9206). HRP-conjugated secondary antibodies and dilutions used were: anti-rabbit 1:8000 (GE Healthcare, NA934), anti-mouse 1:8000 (GE Healthcare, NXA931), anti-rat 1:8000 (GE Healthcare, NA935), anti-chicken 1:10,000 (Invitrogen, A16054). Western blots protein levels were analyzed using Image Lab (Bio-Rad).

## Brain disaggregation, FACS and sorting analysis

Third instar larval brains were processed as indicated in [52]. After disaggregation, cells were immediately separated using FACS. For sorting analysis, cells were separated using BD FACSDiva 8.0.1, then RNA was extracted from sorted cells as mentioned above (see qPCR analysis).

### Imaging of pupae

10 d pupae were imaged using MZ FL III Fluorescence Stereo Microscope (Leica).

### Patients’ samples, cell culture and pharmacological treatment

GBM patients’ samples were obtained from the Neurosurgery Unit of Fondazione IRCCS Ca’ Granda Ospedale Maggiore Policlinico. GBM samples were processed as previously described [10]. All experiments were performed on 3 ATP6V1G1^Low^ and ATP6V1G1^High^ patients. NS were treated for 24 h with BafA1 5 nM and 10 nM (Santa Cruz Biotechnology, sc-201,550).

### Electron microscopy

NS were fixed in 2.5% glutaraldehyde, embedded in 2% agar solution, post-fixed in 1% osmium tetroxide in phosphate buffer, dehydrated and embedded in epoxy resin. Images were captured at 1840X magnification, using an FEI Tecnai G2 20 Transmission Electron Microscope at Alembic – San Raffaele (Milan, Italy).

### Statistical analysis

All experiments were repeated at least three times for quantification and the mean with standard deviation (S.D.) is shown. P-values are as follows: P* ≤ 0.05; P** ≤ 0.01; P***≤ 0.001. Quantifications were performed with ImageJ while GraphPad Prism was used for statistical analyses. Statistical methods are detailed in the figure legends. Source data for all quantified experiments are provided in Table S3.

## Supplementary Material

Supplemental Material
